# The lived experiences of patients undergoing hemodialysis with the concept of care: a phenomenological study

**DOI:** 10.1186/s12882-018-1138-4

**Published:** 2018-11-26

**Authors:** Nahid Shahgholian, Hojatollah Yousefi

**Affiliations:** 1 Critical Care Nursing Department, Faculty of Nursing and Midwifery, Isfahan University of Medical Science, Isfahan, Iran; 2 Nursing and Midwifery Care Research Center, Adult Nursing Department, School of Nursing and Midwifery, Isfahan University of Medical Science, Isfahan, Iran

**Keywords:** Lived experiences, Care, Hemodialysis, Phenomenology, Iran

## Abstract

**Background:**

Providing patient-centered care needs in patients with chronic renal failure undergoing hemodialysis is important in inspiring their confidence to continue their treatment and promote their mental and social health. Therefore, recognizing the concept of care from the viewpoint of these patients can be helpful in designing the care programs of this vulnerable group. Accordingly, the aim of this study was to reveal the meaning and concept of care based on the experience of patients with chronic renal failure undergoing hemodialysis.

**Methods:**

Using a descriptive phenomenological method, this study was conducted on 17 patients who were undergoing hemodialysis. Purposive sampling was performed and data was collected through 30 to 60 min, face-to-face and in-depth semi-structured interviews. Data analysis was performed using Colaizzi’s method.

**Results:**

Seventeen patients (9 women 8 men) aged between 24 and 83, and a minimum of 10 and maximum of 168 months history of hemodialysis participated in the study. After data analysis, 4 themes and 9 sub-themes were extracted, and the concept of care emerged for the participants as empathy, companionship in everyday needs, social support and concern, and good-quality dialysis.

**Conclusions:**

Based on the results of this study, the concept of care from the viewpoint of patients emerged in the form of empathy, companionship in everyday needs, social support and concern, and good-quality dialysis. It is recommended that caregivers of patients consider these concepts in the design of patient-centered care programs.

## Background

Hemodialysis is the most common treatment for the end-stage chronic renal failure in Iran and in the world [1], so that by the end of 2016, the number of patients undergoing dialysis is estimated to be 2,989,000, 89% of which are hemodialysis patients. In Iran, by the end of 2016, the number of patients undergoing hemodialysis has been estimated to be 29,200 [2]. Hemodialysis increases patients’ longevity but, at the same time, imposes many restrictions on these patients and leads to many physical, mental, social and economic complications. Minimizing these complications need a comprehensive care for these patients.

Care is divided into three groups of professional care by the health care team including nurses, home care by the family members, and social care. However, regardless of who provides the care, there is still no specific meaning for this concept which can be used in all situations [3]. Additionally, in spite of the care provided by the health care team, family and society to these patients, they still believe that they don’t receive enough care [4]. The word “care” has many meanings in Persian, such as the provision of what is necessary for the health, welfare, maintenance, protection, attention, guard,, lookout on, and watch of someone. Accordingly, it seems that the point of view of the patients towards this concept is different from the caregivers. Any successful planning and intervention for these patients need to familiarity with this concept based on the lived experience of the hemodialysis patients. The reason is that receiving this concept from the viewpoint of patients can develop the care provided for them and, using this concept, the care team can design a realistic patient-centered care plan and provide an effective intervention.

Many conducted studies have focused on how to care for these patients in order to reduce the complications of disease and treatment, increase the quality of life, reduce stress and improve the mechanisms of compatibility in these patients. However, none of these studies have investigated or considered the viewpoint of patients [5, 6]. Shafiee et al. compared the barriers of blood glucose monitoring in diabetic patients from the viewpoint of patients, healthcare staff and family of the patients. They found significant differences and argued that such difference or disagreement results in the failure of diabetes control in these patients [7].

Atashzadeh et al. also compared the concept of nursing quality in the group of patients, doctors and nurses. In this study, the researchers emphasized the disagreements among these three groups and believed that these disagreements made the healthcare staff not be able to improve the quality of care expected by the patients and, hence, the patients were not satisfied with the quality of the provided care [8]. Because of having a different treatment process, patients undergoing hemodialysis have a different experience of the concept of care. However, studies that investigated the experience of patients undergoing hemodialysis [9, 10] did not cover the concept of care from the viewpoint of the patients and based on their experiences. Accordingly, it seems that conducting a qualitative research in this area, through a deep understanding of the concept of care from the viewpoint of the patients and their experience of care, can help approaching the view of the patients to that of professional, home and social caregivers. It also improve the quality of care, increase the effectiveness of care, provide better services, and promote the patients’ health and quality of life.

Phenomenology is one of the qualitative research methods suitable for understanding the depth of experience and the concept of a phenomenon [11, 12]. According to Thorne (2016), phenomenology is a good method for discovering obscure concepts, including the concept of care, in nursing and other health-related professions [13]. Accordingly, using the descriptive phenomenological method, the researcher decided to investigate the concept of care from the viewpoint and based on the experiences of the patients with chronic renal failure undergoing hemodialysis.

## Methods

Given its purpose of revealing the meaning and concept of care based on the experience of the patients with end-stage renal disease undergoing hemodialysis, this study was a qualitative research with descriptive phenomenological method. This method is used to study experience and describe the concept from the perspective of patients who have lived with the illness, and creates a comprehensive description of the experienced phenomenon in order to achieve an understanding of its essential structure and, beyond its description, provides an interpretation of the phenomenon [11]. Seventeen patients with end-stage renal disease, with purposive sampling, participated in the research. Inclusion criteria: at least three month have elapsed from the start of hemodialysis, no speech and hearing problems, speak fluent Persian, undergo hemodialysis in the hemodialysis unit of Al-Zahra hospital affiliated to Isfahan University of Medical Sciences, Isfahan, Iran, and have a fixed and active medical file in this unit. This unit covers 40 patients permanently. The patients were interviewed one day after dialysis time, in a stable condition, in a room that in an agreement with all patients had been selected next to the dialysis unit. Sampling began from October 23, 2015 and continued to reach saturation, when no new code was extracted, on February 20, 2016.

In order to collect data and access valid and real information, a semi-structured in-depth interview (face-to-face) was used as the main approach. Each interview lasted for 30–60 min.

Each interview started with general questions and was followed up with a calm and flexible format. The interview process actually depended on the respondents’ level of participation. A few questions were used as the interview guide (Table 1). At the same time, some exploring questions such as “could you please explain it more?” or “can you clarify what you mean with an example?” were asked to achieve rich and clear data.

**Table 1:** Interview Questions

What problems hemodialysis has caused to you? What concepts or meanings come into your mind when you hear the word “care”? Whom do you think you need more to take care of you? In what cases do you feel that you are cared for? Who supports you and how? What kind of care makes you feel better? What do you expect from nurses in dialysis unit? How about doctors? Which one of your needs will your family respond to?	

The first researcher then heard each recorded interview several times and transcribed to verbatim and gave a number to each interview. In the same time, using Colaizzi’s seven-step approach, data analysis was performed as follows. 1) The descriptions of the participants were repeatedly read in order to feel them out; 2) 200 important expressions were extracted and numbered; 3) Important expressions were written in scientific language and the meanings were formulated; 4) The constructed concepts/themes were grouped based on their similarity; 5) Nine sub-themes were formed; 6) Similar sub-themes were organized in larger clusters and four main themes were obtained; 7) In order to ensure the accuracy of his/her impressions, the researcher returned them again to the participants, but there was no need to review and repeat the interviews. The researcher reached the data saturation after 17 interviews.

To evaluate validity and reliability of the data, Guba and Lincoln evaluative criteria were used [14]. Accordingly, in order to make the research believable, the review of the co-researchers and participants was also used.

Likewise, to ensure the reliability of the data, after hearing, and analyzing the interviews, the peer review, PhD in nursing, reread and refined the data. The research team used the described methods to minimize the influence of their pre-existing ideas and beliefs on the current research findings.

To provide transferability in this research, the researcher used the full introduction of the research, described the background and stages of the research fully and tried to choose samples in maximum variations.

This research was approved by the Ethics Committee of Isfahan University of Medical Sciences No. 293333. After receiving the necessary permission from the university, the researcher entered the research site. While introducing herself and providing the necessary information to the patients, the researcher explained the purpose and process of the research. After filling out the written informed consent form, the time and place of the interview was determined by an agreement between the participants and the researcher. During the interview, feedback and oral consent were also obtained. Additionally, to maintain anonymity, each interview was given a number. Before beginning the interviews, the participants’ permission was obtained, and they were assured that their names and information would remain confidential. The participants had the absolute discretion to leave the study whenever they wished.

## Results

Seventeen patients (9 female and 8 male) aged between 24 and 83, and minimum of 10 and maximum of 168 months of treatment duration participated in the research. In terms of education, the participants ranged from illiterate to master’s degree; seven were married and their spouses took care of them; and, diabetes was the most common underlying disease causing chronic renal failure (Table 2).

**Table 2:** Demographics’ variables of participants

Variable		frequency	
		Number	Percent %
Sex	female	9	53
	male	8	47
Education	illiterate	4	23.5
	Primary to Diploma	9	53
	College	4	23.5
Married status	Married	9	53
	Single, widow	5	29.4
	divorce	3	17.6
Underlying disease	diabetes	9	53
	Polycystic	2	11.75
	hypertension	2	11.75
	unknown	4	23.5
Duration of dialysis (month)	10–24	9	53
	25–48	5	29.4
	> 49	3	17.6

Analyzing interviews, 200 inferential codes, 9 sub-themes and 4 main themes were extracted. From the perspective of the hemodialysis patients, the care phenomenon was defined through the formation of concepts such as empathy, accompaniment in meeting daily needs, social support and concern, and high-quality dialysis.

The concept of empathy was shaped by the feeling of receiving psychosocial support from the treatment team and emotional support from the family. Help with daily activities and the provision of adequate nutrition were the sub-themes that formed the theme of accompaniment in meeting daily needs. Social support and concern, was another theme that, in explaining the phenomenon of care, was formed by a sense of society’s understanding of the patient’s condition, provision of employment opportunities and financing. High-quality dialysis was another theme which included the sub-themes of meticulous care during dialysis and advanced or unbroken dialysis machine (Table 3).

**Table 3:** Themes and subthemes

themes	subthemes
Emotional support	Psychological support by the treatment team Family emotional support
Accompaniment in meeting daily needs	Assistance for daily activities Provide proper nutrition
social support and concerns	Society’s Understanding of the patient conditions Provide employment opportunities Financing
High-quality dialysis	Careful care during dialysis intact dialysis machine

### Emotional support

Because of numerous dialysis sessions, the participants spent a lot of time with healthcare staff including nurses and doctors. As such, they expected the medical staff to support them psychologically and emphasized their empathy:

‘When nurses listen to me, I’m sure they care for me.’ (Participant 5)

‘When I was hospitalized for my heart condition, I’d like to visit the staff of my own ward (hemodialysis); I was very alone.’ (Participant 7)

Emotional support of the family was another extracted concept and participants stated that emotional support of family members is an integral part of care:

‘I’d like my wife and my children to listen to me and spend more time with me; when they are with me, I’m not afraid of the disease.’ (Participant 3)

What the statements of the participants implied was that the empathy of the health staff, especially the nurses, and the emotional support of family members made the patients feel secure and less worried and, thus, they considered empathy as a concept of care.

### Accompaniment in meeting daily needs

The participants stated that because of their old age, underlying diseases, fatigue and boredom, they often need the help and support of their family for doing daily activities including healthcare activities:

‘I have a blurred vision and can’t see clearly; I can’t shave my face or trim my fingernails; my wife or my children have to do these for me.’ (Participant 10)

Providing an adequate nutrition by the family was another sub-theme of accompaniment in meeting daily needs. Because of their illness, nutritional restrictions are necessary for these patients, and because of their physical conditions, the provision of a special diet requires the collaboration and support of their family:

‘My wife knows which kinds of foods contain phosphorus and potassium, and when it comes to cooking, she is careful and controls my regimen; I can’t do it myself.’ (Participant 2)

‘My family should make me a meal, I can’t do it myself’ (Participant 3)

Participants’ statements indicated that family accompaniment was essential for daily activities such as providing personal health and support in going on a diet. Hence, in the opinion of the participants, accompaniment was one of the concepts of care.

### Social support and concern

Increasing the society’s understanding of the condition of the patients was another theme. The patients expressed their dislike of the pity of others and stated that they wanted others to understand them. Thus, instead of pity that might annoy them, they expected others to help and support them when necessary:

‘People don’t understand us; if they did, they would, for example, give up their seat to us in the bus, or give us they turn in the pharmacy or doctor’s office.’ (Participant 12)

‘Everyone is very busy and there is no support. Many people pity us but I don’t like it.’ (Participant 10)

Providing job opportunities and financing were the sub-themes emphasized by the participants. Because of frequent dialysis sessions, job loss, insufficient ability to work hard and continuously, medical expenses, transportation costs and disproportion between income and treatment or life costs, the patients had economic problems. As such, they believed that having a suitable job and being secured financially is a kind of care:

‘I lost my job because of dialysis; charity doesn't help much; I wish the association helped more and the insurance covered the cost of all drugs; or at least we could have a good job.’ (Participant 3)

Participants’ remarks implied that the society has to change its attitude towards the condition of these patients. As the lack of an organized program to support patients has led to a lot of economic problems for them, providing a job in proportion with the physical condition and dialysis time of these patients can help them benefit from a systematic economic support.

### High-quality dialysis

Since hemodialysis is very sensitive process, patient care and control during dialysis is important. Thus, a meticulous care during dialysis is highly important for the participants:

‘Some personnel don’t set up the machine based on my condition and my blood pressure drops.’ (Participant 7)

‘Every time during dialysis they control my blood pressure five times; I’m afraid of drops in my blood pressure during dialysis; I have spasm in my legs.’ (Participant 13)

An unbroken or advanced dialysis machine was another issue that the participants referred to and stated that a high-quality dialysis is almost impossible without a well-functioning dialysis machine:

‘The machines are broken and don't lose weight well; we’re thirsty between two dialysis; we drink water and get short of breath; our pressure drops under the dialysis; they disconnect us quickly from the machine and going home we are not well.’ (Participant 4)

Accordingly, the participants considered high-quality dialysis as a part of care and emphasized the importance of a meticulous care during dialysis and the proper functioning of the dialysis machines.

Based on the results of the research, the lived experience of dialysis patients shows that care for these patients means empathy, accompaniment in meeting daily needs, social support and concern, and high-quality dialysis. These concepts thus have to be considered in the care plans designed for these patients by the medical staff, domiciliary caregivers and social agents.

## Discussion

The four main themes of emotional support, accompaniment in meeting daily needs, social support and concern, and high-quality dialysis were considered by the participants as the concepts of care; and, it seems that the participants of the present study emphasized the psychological aspects of care more than its physical aspects.

The results of the study conducted by Georgia showed that most hemodialysis patients suffer from a heavy burden of psychological problems [15].

The empathy of the medical staff was one of the concepts of care and the participants emphasized the effectiveness of the relationship with the nurses and doctors and remarked that this relationship can be soothing and reassuring for them. Davison and Simpson believe that the role of personnel in communicating with the patients and their family is very important and can raise their hope. They argue that the nurse’s speaking to the patients about their situation can sometimes be the source of relief and hope for the health and well-being of the patients [16].

The participants’ statements in the present research suggested a significant role of the empathy of the family members in the care and support for the patients. In this regard, a research has shown that family members, especially one’s spouse, have the most important role in providing mental health services to a patient with chronic illness. The patients considered their spouse as the key person in supporting them, that is, the support of the patient’s spouse was the most important source of support during the illness [17]. Asgari et al. also obtained the two concepts of family unification and empathy and responsible accountability of nurses [18], which is in line with the results of this research.

Therefore, it can be concluded that although hemodialysis patients are exposed to mental stressors, empathy and psychological support can help them with these stressors. Empathy from different sources such as family and health care staff can reduce the physical and psychological problems of these patients, thereby helping the patients to cope with the illness more easily, keep away from isolation and gain more vitality and energy.

Accompaniment in meeting daily needs of the patients was another extracted main theme and the participants emphasized that accompanying them with daily activities and providing appropriate nutrition is a concept of care. These patients usually suffer from pain, energy shortages, insomnia and heart condition, and limitation in their physical activity disrupts their physical functioning in such a way that they have difficulty in doing their daily activities [1]. Uremia causes irritability, loss of appetite, insomnia, fatigue, memory loss, impaired judgment and poor concentration and, consequently, these patients sometimes need help in doing their simplest daily tasks [19, 20].

Another need of these patients is the provision of food by the family and based on the prescribed diet. Most of the participants stated that, because of fatigue and decreased energy, they are not able to prepare the recommended food, do not have the incentive to follow the diet, and need the support of their family. Haririan et al. showed that supporting the patients increases their compliance with therapeutic regimen, especially food regimen, thereby improving the quality of life in them [21]. In a qualitative research investigating the barriers of adherence to therapeutic regimen in the patients with type 2 diabetes, the participants have stated that one of the factors impeding the compliance with therapeutic regimen has been inadequate family support [7].

From the statements of the participants and the mentioned studies it can be concluded that accompaniment in meeting the daily needs of the patients is a very important factor that should be considered in designing a care plan for these patients.

From the viewpoint of the participants, support through increasing the society’s understanding of the patients’ condition, providing appropriate jobs and financing were other care-related concepts. The type of the support and the patient’s perception of it contribute to its effectiveness, so that the participants stated that they dislike pity and expect others to understand their conditions, and believed that effort to achieve this goal is related to the concept of care. Siegert et al. found that dialysis exerts a pressure on the patients and their family, but the reaction of others can have a significant impact on their mental status [22]. Therefore, it seems that changing society’s attitudes and understanding towards these patients would help to support these patients instead of pity them.

The necessity of employment and financing were two other sub-themes extracted from the statements of the participants and emphasized by most of them. As these patients spend a considerable amount of time doing dialysis and medical care and are often not in a good condition, they often encounter many limitations with regard to their employment, lose their jobs and have many economic problems. Accordingly, they expect the government and the association for the support of kidney patients to provide the ground for their employment and believe that such a support is one of the concepts of care. Rafiee and Rambod also showed that many hemodialysis patients had lost their jobs and experienced many economic and social problems and were unable to provide some of their own and their family needs [23]. However, it should be noted that financial problem of the patients is not specific to our country, as Hui-Dan et al. also found that most hemodialysis patients have a lot of financial problems [24].

The support of social organs for patients, especially economic support, reduces the problems of these patients and plays an important role in tolerating the disease. Most of the participants of this study were satisfied with the support of social organizations, but did not consider it enough and expected more support, especially more financial support [9]. Therefore, designing a social support program, including economic support for these patients seems to be essential.

High-quality dialysis was another extracted main theme and, in this regard, a meticulous care during dialysis and using a well-functioning dialysis machine were emphasized by the participants. They stated that care during dialysis, including minute adjustment of the dialysis machine, blood pressure control, precise weight control and having sufficient skill in dialysis, is of particular importance. Kaba et al. wrote that patients undergoing hemodialysis always suffer an anxiety caused by possible problems and the likelihood of death during dialysis and, hence, dialysis nurses should have the sufficient knowledge and skill to prevent such problems [4].

The nurse should have enough information about the patient and the dialysis machine in order to implement a safe and high-quality dialysis program [25]. Therefore, meticulous care during dialysis was considered by the participants to be essential. The participants complained about broken machines, their frequent alarms and their inability to lose weight and believed that the existence of unbroken dialysis machines is the proof of a high-quality dialysis. Dialysis machine is an integral part of treatment for these patients and the proper function of the machine can directly affect the outcome of the treatment and complications of the dialysis [26]. Thus, according to the participants, high-quality dialysis was one of the concepts of care that should be considered in the design of a care program for these patients.

Although qualitative research gives us a deep understanding of the phenomenon, because of the expansion of different topics, one cannot get all the dimensions of a topic in interviews with a limited number of individuals. Accordingly, revealing new themes is likely only through conducting more interviews with a diverse sample of participants not captured in this study. Therefore, although data saturation in this research was reached, additional participants in future studies may address more dimensions of the concept of care.

## Conclusions

According the findings, we can answer the research question that “what is the lived experience of hemodialysis patients with the concept of care?” From the viewpoint of the patients, empathy, accompaniment in meeting daily needs, social support and concern, and high-quality dialysis constitute the concept of care. One of the unique results of the present study, compared with other similar ones, was the emphasis of the participants on the mental aspects of care. Accordingly, these aspects have to be considered in the care plans designed by the healthcare team for these patients.

## Abbreviation

PhD: Doctor of Philosophy
